# Medical education and the healthcare system – why does the curriculum need to be reformed?

**DOI:** 10.1186/s12916-014-0213-3

**Published:** 2014-11-12

**Authors:** Gustavo A Quintero

**Affiliations:** School of Medicine and Health Sciences, Universidad del Rosario, Carrera 24 # 63C-69, Bogota, Colombia

**Keywords:** Health-illness process, Health system based curriculum, Transformative education

## Abstract

Medical education has been the subject of ongoing debate since the early 1900s. The core of the discussion is about the importance of scientific knowledge on biological understanding at the expense of its social and humanistic characteristics. Unfortunately, reforms to the medical curriculum are still based on a biological vision of the health-illness process. In order to respond to the current needs of society, which is education’s main objective, the learning processes of physicians and their instruction must change once again. The priority is the concept of the health-illness process that is primarily social and cultural, into which the biological and psychological aspects are inserted. A new curriculum has been developed that addresses a comprehensive instruction of the biological, psychological, social, and cultural (historical) aspects of medicine, with opportunities for students to acquire leadership, teamwork, and communication skills in order to introduce improvements into the healthcare systems where they work.

## Background

### From the Flexner report to today

Abraham Flexner, in his famous 1910 report [1], proposed the model of medical education that prevailed during the first half of the 20^th^ century. However, 15 years after his report, Flexner himself recognized that this new medical curriculum gave precedence to the scientific aspects of medicine over its social and humanistic aspects [2]. Since then, medical education has been the subject of on-going debate. The core of the discussion revolves around the importance of scientific knowledge on biological understanding at the expense of its social and humanistic characteristics. The chronological evolution of medical education models is summarized in Table 1 [3-8]. With globalization and the idea to implement strategies to promote global health, a number of medical schools have taken up the challenge of modifying their curricula in order to educate physicians capable of responding to the current and future trends arising from population health maintenance and the consequent practice in that context. These changes aim to ensure integration between basic and biomedical sciences with clinical sciences and to reduce unnecessary knowledge overload through a new study plan for medicine.

**Table 1:** **Chronological evolution of medical education models**

**1910**	Abraham Flexner proposed a curriculum with biological model that prevailed during the first half of the 20^th^ century.
**Mid-1950s**	Hugh Rodman and E. Gurney Clark published “Preventive Medicine for the Doctor in His Community”, which put forward the concept of a natural history of disease, supporting the idea of preventive medicine as an alternative for physicians to understand individual and community health-illness problems.
**1974**	H.L. Blum and Marc Lalonde introduced the model of health fields, where health-illness process depended on four groups of factors (genetics, behaviour, health services and the environment).
**1978**	Alma Ata Conference adopted the global strategy of Health for All where the focus of medicine was health promotion and illness prevention, and medical schools initiated processes to adapt their curriculum to these schemes.
**1986**	The Ottawa Charter, signed at the international conference adopting health promotion as a new approach in healthcare in order to overcome the shortcomings of the previous models.

This approach continues to be based on a biological perspective of the health-illness process and the need to comprehensively incorporate into it the socio-humanistic and population health science fields has not been foreseen. In the few instances where this has been performed, incorporation has been limited to the introduction of isolated issues, without an organic connection with the overall curriculum or tying them in as auxiliary elements in public health sciences and risk factors in preventive medicine. Scientific knowledge and skills competences still hold their supremacy over delving into the dimensions of the human being, necessary for the development of socio-humanistic competences.

### Health-illness process

Characterization of the health-illness process is a crucial step prior to medical curriculum design. This will determine the understanding of the reality of health and illness of communities and individuals, and the action that should be taken to prevent disease and restore and maintain health. Thus, the new curriculum will provide better professional training in order to address and intervene the specific healthcare needs.

Curriculum design should then consider health and illness not as states but rather as processes resulting from the interaction of multiple forms of determination that operate simultaneously in the ambit of individuals, collectives, and in society and culture, all of which have a historical character. In fact, society and culture are not causal factors as imagined in positivist epidemiology, but are the broad and general continents where health and disease occur.

The health-illness process is, in consequence, primarily a social and cultural process where the biological and the psychological is subsumed and is socially and culturally determined [4].

### Medical education based on the healthcare system

Physicians should be prepared to address complex systems and to lead such systems in an effort to protect the best interests of patients and communities. This reality makes it necessary to modify the way medicine is taught and learned; this is not just a matter of schooling in basic and clinical sciences. It is crucial to introduce socio-humanism and population health sciences (healthcare system) into the teaching of medicine, in an integrated manner, as well as to provide opportunities for students to train in teamwork, communication, and professionalism in order to be able to practice in an uncertain profession such as medicine. An uncertain profession is one where the professional cannot directly control the outcome of his work [9].

One hundred years after the Flexner report, The Carnegie Foundation for the Advancement of Teaching, the same organization that sponsored his study, conducted an investigation on medical education. Based on this study, four goals for modern medical education were recommended (Table 2) [10] and a new generation of curriculum reform proposed. As much as Flexner introduced medicine to science, the advent of Problem Based Learning (PBL) made a change in the didactic technique; now, the System Based Curriculum, should “*improve performance of the healthcare system in adapting core professional competencies into specific contexts, on the basis of global knowledge*”. In those three generations of reforms, medical education has moved from informative learning that produced expertise, to formative learning that produced professionals, to transformative learning that is “*about developing leadership attributes; its purpose is to produce an enlightened change agent*” [10].

**Table 2:** **Goals for modern medical education**

1.	Standardize the learning outcomes and general competencies and provide options for customizing the learning process, providing opportunities for experiences in research, policy making, education, etc., reflecting the broad role played by physicians.
2.	In practice, physicians must constantly integrate all aspects of their knowledge, skills and values. They should acquire skills to educate, advocate, innovate, investigate and manage teams.
3.	Medical schools and teaching hospitals should support the engagement of all physicians-in-training in inquiry, discovery and systems innovation.
4.	Development of professional values, actions, and aspirations should be the backbone of medical education.

The study also considered interdependence in education as a key element in a systems approach because it underscores the ways in which various components interact with each other, necessary to provide inter-professional education that promotes collaborative practice.

### The Rosario experience: changing the curriculum to enhance the healthcare system

Colombia is a country with 48.3 million people and an upper middle-income level as defined by the World Bank [11], with a life expectancy at birth of 74 years. Three years ago, the government issued a law focusing the attention of healthcare to primary care [12]. In Colombia, an increasing number of medical schools have implemented curriculum reforms to be in tune with the times but, perhaps, most of them have not been addressed to produce change agents which will satisfy the needs of the healthcare system.

According to Garcia [13], “*medical education is the process for training doctors, subordinate to the dominant economic and social structures in societies in which it takes place*”. Therefore, medical education cannot be divorced from social reality. If medical education is a process, it must be understood as a continuum that begins with undergraduate training but does not end there; it is lifelong learning. Such learning must also seek the welfare of the society where it will be implemented, which in a globalized world, is universal [14].

We have long been discussing the major changes required in the healthcare system and the reforms to be undertaken to achieve them. However, if substantial changes to medicine teaching methods are not introduced, these will not be obtained.

**Figure 1:**
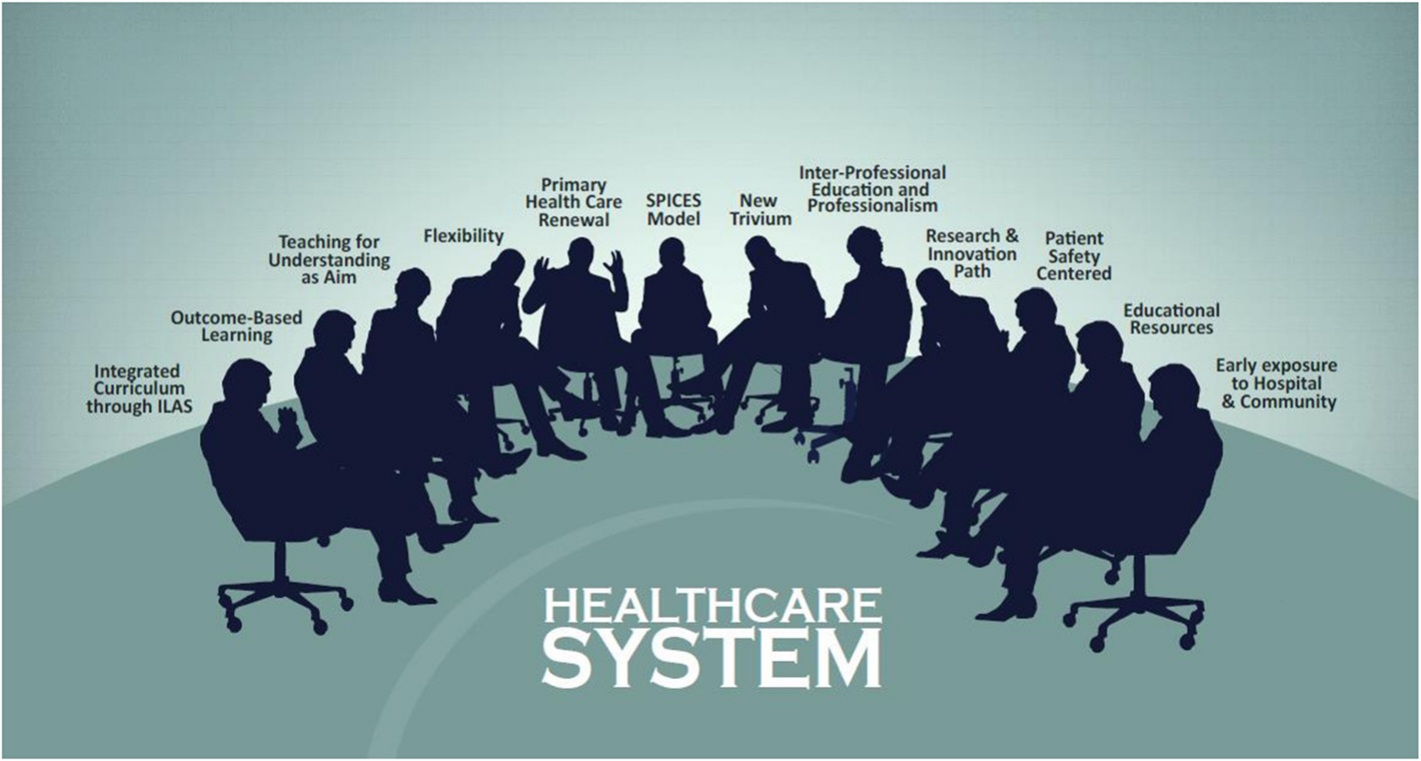
**Curriculum attributes facing the healthcare system.** Medical education undergraduate curriculum at the Rosario University. The curriculum´s key characteristics are depicted including those allowing opportunities for students to acquire leadership, teamwork and communication skills in order to deal and introduce improvements into the healthcare systems where they will work. (Original source) Abbreviations: ILAS: Integrative learning activities by system, SPICES: student-centred/teacher-centred, problem-based/information-gathering, integrated/discipline-based, community-based/hospital-based, elective/uniform and systematic/apprenticeship-based.

Commencing in 2013, staff at the Rosario University School of Medicine and Health Sciences, Bogotá, Colombia, implemented an undergraduate curriculum reform in medicine which has implicit variations based on the healthcare needs (Figure 1), with curriculum attributes for undergraduate medical education in relation to the healthcare system.

The curriculum integrates basic/biomedical, clinical, socio-humanistic, and population health sciences through a teaching and learning method termed Integrative Learning Activities System-based, a PBL variant, with opportunities for students to acquire leadership, teamwork, and communication and professionalism skills, in order to introduce competences to improve the healthcare system where they will work [15]. Teaching in an integrative way allows doctors to practice consequently. The curriculum is supported on learning outcomes and its aim is Teaching for Understanding [16], a non-memory method for teaching and learning to cultivate learners’ capability to think creatively, formulate and solve problems, and collaborate in generating new knowledge. We adopted the SPICES model proposed by Harden et al. [17] with learning focus in early expositions, in both hospital and community settings to benefit primary care in its conception of Primary Health Care Renewal striving for integration of all levels of healthcare.

The curriculum has a 30% flexibility based on elective/selective components with three graduation scenarios: MD, MD with possibilities to pursue an MSc degree, or as MD with options to obtain a second undergraduate title. All this in order to provide opportunities for students to obtain experience in research, policy making, education, primary care, and other areas, reflecting the broad role played by physicians and the Colombian societal needs.

The medical curriculum, as a part of the School of Medicine and Health Sciences, which has another five programs in health science areas, favors interprofessional education to provide enough skills for collaborative practice and professionalism, which is the main objective of the school, understood as value-centered education. Communication skills are taught under the “new Trivium” conception that provides skills on learning how to learn and acquire cognitive-linguistic competences that make communication a way to manage learning [18]. Communication is also basic for humanism in medicine. The curriculum also introduces a path to research and innovation through basic/biomedical sciences and clinical sciences (translational medicine), the socio-humanistic sciences, and population health sciences. Another route refers to patient safety, which may contribute to decreasing errors and improving quality in medical practice.

All of the above are supported by educational resources such as Information and Communication Technologies through the “Mutis electronic platform” and the Mentor program, a healthcare integrated network that includes two university hospitals and a primary care setting which allow an early exposure to the clinical environment.

With the new curriculum, we expect to educate doctors who may lead healthcare system changes that positively affect the wellbeing of individuals and the communities where they work. We aim for a true transformative learning experience in a lower middle-income country.

## Conclusions

Medical education calls for a profound change in the way it is taught and learned – a change which provides the welfare society needs, wherever a doctor practices. Where a doctor can be recognized as a leader capable of introducing such a transformation in an unstable world in which previously controlled diseases re-emerge and new ones arise, population aging increases at a rapid pace, systems face health coverage problems and increasing chronic diseases, and health public policy consumes much of our nations’ gross domestic product.

Medical education must be based on a healthcare system with global thinking and local implementation in an interconnected world. The reform of undergraduate medical curricula should follow this guide in order to contribute to the medical mission. Our experience shows that it is possible to make curriculum changes in medical programs consistent with the current societal needs – a medical education based on the healthcare system.

## Abbreviation

PBL: Problem based learning
